# Development of a Dementia Intensive Advanced Practice Provider Fellowship

**DOI:** 10.1002/alz.087438

**Published:** 2025-01-09

**Authors:** Anne K Arthur, Lindsey Gillen, Samantha Fikru, Jessica Malcolm, Eric D Vidoni

**Affiliations:** ^1^ University of Kansas Medical Center, Kansas City, KS USA

## Abstract

**Background:**

The aging population is driving an unprecedented increase in the number of individuals with Alzheimer’s disease and related dementias (AD/ADRD). Currently, there is limited availability of specialists in AD/ADRD and a growing need in the United States for new access points to treat the estimated 7.2 million people with AD/ADRD expected in 2025. Care access disparities are particularly apparent in the Central Plains states, most of which are considered “Neurology Deserts”. In response, the University of Kansas has developed an Advanced Practice Provider (APP) Brain Health Fellowship to increase sustainable access to AD/ADRD care.

**Methods:**

The KU Alzheimer’s Disease Research Center (KU ADRC) and the KU School of Nursing developed a 6‐month AD/ADRD fellowship to train APPs to recognize, diagnose and treat AD/ADRD. Learning modules and rotations were selected by an expert panel, including our neurology and APP clinical fellowship directors, and cover relevant topics and departments encountered during the diagnosis and treatment of AD/ADRD. Examples include AD/ADRD treatments, neuropsychology, neuroimaging, biomarkers, social work support, end of life care planning, and research. The weekly schedule includes didactics, precepted clinics, mentored clinics, and specialty clinics. The program director and fellow review evaluations, clinical progress, and career planning monthly. The fellow completes pre‐ and post‐fellowship competence and dementia knowledge surveys developed by the program. The fellow receives a competitive salary, laptop, and education materials.

**Results:**

To date, one fellow has completed the program. Pre‐ and post‐test results showed an 85% increase in AD/ADRD knowledge. The fellow, on average, rated the preceptors and program at “Above Average” to “Excellent”. Feedback during and after the fellowship has been incorporated into ongoing programming. The first fellow was hired by the KU ADRC.

**Conclusions:**

The KU ADRC APP Brain Health Fellowship is a first‐of‐its‐kind approach to develop clinicians trained to care for the growing number of individuals with AD/ADRD. Our preliminary results suggest the fellow increased AD/ADRD care competence and the program was valued by the fellow. The immediate post‐program employment of the fellow is evidence of potential benefit to increasing access to care. The fellowship will continue in 2024 and train two more fellows.

